# Number of translations and translation direction in masked translation priming: evidence from unbalanced English–Chinese bilinguals

**DOI:** 10.3389/fpsyg.2024.1500750

**Published:** 2024-11-27

**Authors:** Quanbei Zhao, Jia Li, Wenxin Xiong, Hongbing Xing

**Affiliations:** ^1^School of Chinese Language and Literature, Beijing Foreign Studies University, Beijing, China; ^2^Faculty of Foreign Studies, Beijing Language and Culture University, Beijing, China; ^3^School of International Media, Jilin International Studies University, Jilin, China; ^4^Institute on Educational Policy and Evaluation of International Students, Beijing Language and Culture University, Beijing, China

**Keywords:** masked translation priming, number of translations, translation direction, unbalanced English–Chinese bilinguals, bilingual mental lexicon

## Abstract

Within the research field of bilingual lexical representation and organization, much attention has been given to whether two languages share a conceptual system and what factors modulate the connection between this conceptual system and the lexical systems of a bilingual’s L1 and L2. One of the most valid ways in the psycholinguistics domain to explore these doubts is to examine the masked translation priming effect and the priming asymmetry through cross-language priming experiments. In this study, a masked priming lexical decision task was conducted with unbalanced English-Chinese bilinguals to investigate whether the masked translation priming effect exists in both translation directions, and to further reveal how the number of translations, which can be categorized into one-translation pair and more-than-one-translation pair conditions, affects the priming effects and modulates translation priming asymmetry. It was demonstrated that both translation direction and the number of translations influence the priming effect. Specifically, the priming effect was observable from L1 to L2 but not from L2 to L1, and the priming for one-translation pairs was significantly greater when compared to that for more-than-one-translation pairs. Moreover, the impacts of translation direction on the priming effect differed between the one-translation pair and more-than-one-translation pair conditions: under the former condition, substantial priming occurred in both directions, whereas for the latter condition, it was observed only in the L1–L2 direction. Several models of the bilingual mental lexicon, mainly the Revised Hierarchical Model and the Distributed Conceptual Feature Model, were used to elucidate the above results.

## 1 Introduction

The field of psycholinguistics has recently seen significant attention to the organization and access of bilingual memory (Ferré et al., 2023). A primary concern in the studies of bilingual lexico-semantic representation and organization has been identifying factors that influence the links between the lexical systems of a bilingual’s two languages and the conceptual system (Jiang, 2023). Related studies have spanned a range of experimental paradigms, including translation priming, picture-word presentation, and language switching paradigms (Bailey et al., 2024), along with diverse experimental tasks, for instance, lexical decision task (Lee et al., 2018; Smith et al., 2019; Ferré et al., 2023; Scrimshire et al., 2023), semantic categorization task (Wang and Forster, 2010; Tytus, 2017; Lijewska et al., 2018; Yang et al., 2024), and naming task (Broersma et al., 2016; Dylman and Barry, 2018; Ramanujan and Weekes, 2020; Momenian et al., 2021, 2024). The translation priming paradigm is one of the most feasible approaches for investigating such questions, serving as a crucial methodology for exploring the underlying mechanisms of the bilingual mental lexicon (Lee et al., 2018; Chaouch-Orozco et al., 2024).

The translation priming effect is typically evident in a faster mean reaction time (RT) in the case where the prime and the target are translation equivalents mutually, relative to a cross-language unrelated pair (Chaouch-Orozco et al., 2023; Scrimshire et al., 2023). The translation priming paradigm, applied in research on bilingual lexical representation and organization, can be broadly classified into two categories: masked and unmasked priming (Chen et al., 2020). The masked priming translation paradigm is more frequently utilized than the unmasked one (Smith et al., 2019). The use of masked primes greatly reduces participants’ likelihood of adopting intentional strategies, since they are not even aware of the subliminal prime, let alone identify the prime-target relationship. As a result, the masked priming paradigm is widely regarded as providing a more solid basis on understanding the unconscious and automatic semantic activation throughout the early processes involved in bilingual word recognition (Qiao and Zhang, 2017; Lim and Christianson, 2023; Li et al., 2024). Under such context, the masked translation priming effect is thought of as being a vital proof in supporting the existence of a conceptual system that is shared across a bilingual’s both languages (Ferré et al., 2017; Bu and Lu, 2021; Wang X. et al., 2023), and is also regarded as evidence for non-selective lexical access (Wen and van Heuven, 2017a; Lim and Christianson, 2023; Scrimshire et al., 2023).

Lexical representations in one language may impact lexical access in another language. However, there is still no consensus on whether cross-language influence exerts facilitation or interference effects in lexical organization and access. The existence of cross-language priming effects reflects facilitation, but whether and to what extent these effects occur can vary depending on the translation direction (Bailey et al., 2024). Therefore, among a number of factors affecting the masked translation priming effect, translation direction has drawn researchers’ considerable attention. Nevertheless, bilinguals who process different-script language pairs, particularly English-Chinese bilinguals with English as their L1, are still under-examined in such studies. As noted by Wen and van Heuven (2017b), Davis and Kim (2021), and Jiang (2023), only Wang (2013) has specifically examined this bilingual group. More importantly, translation equivalent word pairs have both shared and separate conceptual nodes. Hence, the English and Chinese words in any given word pair are not always mapped in a one-to-one fashion (Yang and Wang, 2023; Zhao et al., 2023). However, previous research has rarely examined the way in which the number of translations influences the masked translation priming effect and its modulation of priming asymmetry. The number of translations, as a key factor, has not been addressed in the comprehensive summaries of relevant studies by Wen and van Heuven (2017b), Davis and Kim (2021), or Jiang (2023). Therefore, this study intends to tackle these gaps in the literature. Giving attention to different writing systems, this study provides new empirical evidence from English-Chinese bilinguals to gain further insights into the cross-language priming effect. Additionally, this study contributes to the field by expanding the research scope through directly examining the impact that the number of translations exerts on the masked translation priming effect. It represents, as per the best of our knowledge, the first exploration of how the number of translations impacts the masked priming effect, along with modulating the priming asymmetry for unbalanced English-Chinese bilinguals.

### 1.1 Translation direction in masked translation priming

The cross-language priming asymmetry, which results from comparing the effects of prime type in two translation directions or exploring the way prime type interacts with translation direction, is indicative of an assumption—the sharing of conceptual representations across languages occurs in an incomplete way (Chen et al., 2017).

In past research on the cross-language priming effect, unbalanced bilinguals with a dominant L1 have been the primary participants examined via visual lexical decision tasks (Chaouch-Orozco et al., 2021; Jiang, 2023). In extensive studies reporting cross-language priming asymmetry, some observe priming effects solely in the L1–L2 direction (Dimitropoulou et al., 2011a; Voga, 2020; Chaouch-Orozco et al., 2021; Scrimshire et al., 2023; Tomaz et al., 2024; Li et al., 2024, experiment 1), demonstrating the asymmetry reflects in the direction of translation. While, some others have also found the masked translation priming effects in the L2–L1 direction (Schoonbaert et al., 2009, experiment 2; Lijewska et al., 2018, experiment 1; Davis and Kim, 2021; Chen et al., 2022; Lim and Christianson, 2023), and the priming magnitude is generally smaller in the direction of L2–L1. It indicates the asymmetry is reflected in the priming size as well. In brief, the evidence in regard to the priming effect in the L2–L1 direction for unbalanced bilinguals remains inconclusive and thus warrants further investigation.

Meanwhile, the bulk of research mentioned above has concentrated on bilingual lexical representations within the same language family, for example, Polish-English, Spanish-English, Portuguese-English, etc. (Lijewska et al., 2018; Chaouch-Orozco et al., 2021; Tomaz et al., 2024), with not only insufficient attention paid to languages of different language families, but also inconsistent findings (Chen et al., 2020). Moreover, bilingual participants in studies that have focused on different language families and writing systems, concretely the bilinguals speak both Mandarin and English, tend to be Chinese individuals who learn English under the foreign language education model in China. Learners under such a learning model are typically exposed to considerable vocabulary input in a traditional and formal classroom context. In contrast, there is limited research on L1 English speakers with Chinese learning experiences, whose Chinese learning is usually accomplished through immersion in China after high school. There are certain disparities in the L2 exposure and learning experiences between these two kinds of bilinguals. To date, only Wang (2013) has tested Chinese learners whose L1 is English. Participants in Wang’s study were unbalanced English-Chinese bilinguals with English as their dominant language who resided in Singapore, a bilingual setting where Chinese and English are regularly spoken. During their primary and secondary schooling, participants systematically learned Chinese as a compulsory subject in a structured educational setting. They were required to achieve passing scores on Chinese language proficiency exams needed for university admission. In Wang’s study, an interaction effect between prime type and translation direction was observed in both RT and error rate analyses. Specifically, in the L1–L2 direction, RTs were significantly shorter and error rates were significantly lower, for targets preceded by their translation primes compared to those preceded by unrelated primes. However, in the L2–L1 direction, no significant differences were found between these priming types in either RTs or error rates. In other words, a significant masked translation priming effect was present in the L1–L2 direction, but absent in the reverse direction. These results suggest a cross-language priming asymmetry in lexical decision tasks for unbalanced English-Chinese bilinguals.

### 1.2 Number of translations in masked translation priming

Apart from translation direction, researchers have also uncovered other factors inducing the cross-language priming effect, for instance, L2 proficiency (Duñabeitia et al., 2010; Dimitropoulou et al., 2011b; Lim and Christianson, 2023), stimulus onset asynchrony (SOA) (Schoonbaert et al., 2009; Ferré et al., 2017; Lee et al., 2018), and the number of items per condition (Wen and van Heuven, 2017b). In contrast with these external methodological factors pertaining to the experiment design and execution, factors associated with the intrinsic word characteristics, such as the number of translations, remain to be further explored.

Despite most studies investigating the translation priming effect having picked one-translation pairs (OTPs) as their experimental materials (Schoonbaert et al., 2009; Smith et al., 2019; Chen et al., 2022), more-than-one-translation pairs (MTOTPs) frequently occur in cross-language contexts (Schwieter and Prior, 2020; Deng et al., 2024). Wang and Zhang (2013) zoomed in on MTOTPs, revealing significant priming effects in both directions among proficient Chinese-English bilinguals. Some other studies did not strictly control the number of translations in the stage of material preparation. To prevent error rates from increasing caused by MTOTPs, Tytus and Rundblad (2016) excluded them during data analysis. Ferré et al. (2017) rechecked and confirmed all the targets in the experimental materials as multiple translation words to rule out the involvement of the number of translations as an influencing factor of experimental results. McPhedran and Lupker (2021) matched experimental materials without giving thought to the number of OTPs and MTOTPs, but instead turned to the translation uniqueness score as a covariate in supplemental analyses to examine whether the number of translations affected the results. As previously discussed, existing studies have either considered the number of translations as a control variable, using only OTPs or MTOTPs as experimental materials, or have failed to adequately control for the number of translations, with resulting uncertainties then subjected to item discard or supplemental analyses.

Nevertheless, a handful of studies have inspected how the number of translations affects the lexical processing through the tasks of translation recognition or production, concluding that participants processed OTPs more quickly than MTOTPs (Tokowicz and Kroll, 2007; Laxén and Lavaur, 2010; Basnight-Brown and Altarriba, 2016; Wang and Wang, 2019; Basnight-Brown et al., 2020). Although these studies found that the number of translations significantly affected RTs, they did not factor in the masked translation priming effect in their examination, thereby preventing the direct investigation into how the number of translations impacts the masked translation priming effect.

### 1.3 The current study

To summarize, the impact of translation direction on the masked translation priming effect among English-Chinese bilinguals remains underexplored, and further research is needed to extend and substantiate relevant findings. Furthermore, there is a notable lack of literature directly exploring how the number of translations affects this priming effect and whether the influence of translation direction on the priming effect is modulated by the number of translations. To address the identified research gaps, this study was conducted with unbalanced English-Chinese bilinguals, utilizing OTPs and MTOTPs as experimental materials. It investigated how translation direction and the number of translations influence the masked translation priming effect by means of a lexical decision task within the masked priming paradigm.

One of the models of the bilingual mental lexicon, which emphasizes differences in the strength of connections between lexical and conceptual systems across translation directions, is the Revised Hierarchical Model (RHM) (Kroll and Stewart, 1994; Kroll and Tokowicz, 2001). This model not only postulates the co-existence of two types of connections—lexical and conceptual, but also emphasizes that the lexical connections in the L1–L2 direction are weaker compared to those in the reverse direction. Moreover, L1 words are more strongly connected to the conceptual system in comparison to L2 words.

The Distributed Conceptual Feature Model (DCFM) (de Groot, 1992; van Hell and de Groot, 1998) makes a point of the role of semantic overlap in cross-language processing. This model posits that the meanings of words are stored as nodes at the conceptual level, with lexical nodes linked to a network of distributed semantic features. It assumes variation in the extent of conceptual representation shared by words across languages. Within the model, the priming effect is contingent upon the degree to which semantic features are shared between the words from L1 and L2 (Duyck and Brysbaert, 2004; Schoonbaert et al., 2009). In detail, a greater degree of shared semantic features between words means larger conceptual overlap, which in turn results in a stronger priming effect. The degree of semantic overlap between a word in the source language and its translations in the target language usually differs between OTPs and MTOTPs, with words from OTPs potentially exhibiting a higher degree of semantic overlap.

The two models of the bilingual mental lexicon outlined above serve as a foundation for the research questions articulated in this study, which are as follows:

1. How does translation direction affect the masked translation priming effect?
2. How does the number of translations influence the priming effect?
3. How do the impacts of translation direction on the priming effect differ under OTP and MTOTP conditions?

Based on the aforementioned research questions, three hypotheses were formulated as follows:

*Hypothesis 1*: If the translation priming effect arises through the conceptual route (Ferré et al., 2017; Bu and Lu, 2021; Wang X. et al., 2023), as predicted by the RHM, the priming effect is expected to be more readily observed in the L1–L2 direction instead of the reverse direction. This is attributed to a stronger association of L1 words with conceptual systems. As L2 proficiency improves, the connections between L2 words and the conceptual representation also strengthen. Accordingly, for highly proficient or balanced bilinguals, a significant priming effect may emerge in the direction of L2–L1. For the unbalanced bilinguals in this study, however, the priming effect is more prone to be exhibited in the direction of L1–L2, with its magnitude greater than that in the opposite direction.

*Hypothesis 2*: Given that each word in an OTP has only one translation, the semantic features shared between words in OTPs are more extensive than those in MTOTPs. In accordance with DCFM, larger conceptual overlap typically results in a more pronounced priming effect. If this prediction holds true, we anticipate that the magnitude of priming for OTPs to surpass that for MTOTPs.

*Hypothesis 3*: Likewise, the varying degree of semantic overlap and the larger number of translations may impede participants from processing MTOTPs. This underscores the advantage of a richer semantic representation in the L1 compared to the L2. Therefore, it is reasonable to predict that the influence which translation direction imposes on the masked translation priming effect will vary with the change in primes and targets from OTPs to MTOTPs. Specifically, for MTOTPs, a significant priming effect is more probable to be found in the L1–L2 direction, whereas for OTPs, such an effect could be evident in both directions.

## 2 Materials and methods

### 2.1 Participants

An *a priori* power analysis was performed employing G*Power 3.1 (Faul et al., 2007) with a medium effect size (*f* = 0.25) (Cohen, 1988) and an alpha level of 0.05. The decision to choose a medium effect size was guided by previous studies, including Wen and van Heuven (2017b), Wu et al. (2024), and Deng et al. (2024). The results suggested that 23 participants would be necessary to achieve a statistical power of 0.95. Twenty-four participants were recruited in our experiment. Specific information can be seen in Table 1. Data collection occurred from December 5, 2023, to January 17, 2024, and again from April 22 to 25, 2024, with a temporary pause during a winter holiday.

**Table 1:** Participants’ language background information.

Measure		Value
Number of participants (gender)		24 (16 males)
Age		25.21 (5.29)
Cloze test scores		23.88 (2.67)
Self-rating scores	L1 listening proficiency	6.83 (0.37)
	L1 speaking proficiency	6.83 (0.37)
	L1 reading proficiency	6.75 (0.52)
	L1 writing proficiency	6.63 (0.70)
	L2 listening proficiency	5.50 (0.82)
	L2 speaking proficiency	4.79 (1.00)
	L2 reading proficiency	5.13 (0.97)
	L2 writing proficiency	4.17 (1.14)
	Overall L1 proficiency	6.76 (0.45)
	Overall L2 proficiency	4.90 (0.76)

The overall proficiency refers to the mean scores across the four language skills of the participants.

Prior to the formal experiment, we carefully inquired about and confirmed the participants’ native and second languages, with all participants identifying themselves as English-Chinese bilinguals. Among these, three participants reported having an additional native language besides English: one with Russian, one with Urdu, and one with Spanish. Most of them are pursuing undergraduate or graduate degrees in Chinese language-related majors or are enrolled in advanced Chinese language training programs at several universities in China, predominantly in Beijing. The remaining small portion has already graduated and entered the workforce. The majority of them have passed the HSK (Chinese Proficiency Test) Level IV, while a few who have not taken the test are also engaged in intermediate or advanced Chinese language classes.

Participants self-rated their language proficiency across the skills of listening, speaking, reading, and writing on a 7-point scale (1 for “very poor,” 7 for “excellent”). The paired-samples t-test results manifested that the self-assessment scores for English (L1) skills and overall proficiency were significantly higher than those for Chinese (L2) skills and overall proficiency [all *p*s < 0.001, *Cohen’s d*(s) ≥ 1.48], indicating that the participants were unbalanced bilinguals. Given that a few participants had not taken the HSK test, we took a reference from Wang Y. et al. (2023) to administer a 30-item fixed-ratio cloze test in Chinese with a full score of 30 (Feng et al., 2020). The participants’ average score was 23.88 (*SD* = 2.67). Based upon the criteria set up by the test co-developers (Feng and Zhang, 2023), participants in our experiment were intermediate-advanced Chinese L2 learners. On the whole, our participants were categorized as unbalanced intermediate-advanced English-Chinese bilinguals. All participants had normal uncorrected or corrected vision and were compensated with 70 RMB or 9 GBP for any inconvenience and time spent at the end of their involvement in the experiment.

### 2.2 Research design

We initially drew on experimental materials from prior studies (e.g., Zhao and Li, 2013; Basnight-Brown et al., 2020; McPhedran and Lupker, 2021) and consulted the Vocabulary List from the *Chinese Proficiency Grading Standards for International Chinese Language Education* (Center for Language Education and Cooperation, 2021) to guarantee that the L2 Chinese words selected were appropriately controlled for difficulty and familiarity. In this way, 313 English-Chinese translation pairs were prepared, with the majority of Chinese words comprising elementary and intermediate Chinese vocabulary, for example, “result”–“结果” and “style”–“风格.”

Then, referring to Basnight-Brown et al. (2020) and Lee et al. (2023), we adopted the “first translation” method to ascertain the specific number of translations of all the prepared Chinese and English words. Sixteen English-Chinese bilinguals, who were not invited to take part in the formal priming experiment, were recruited in advance for the translation task. Evenly divided between the two translation directions, they were instructed to record the first translation that occurred to them for each given Chinese or English word. When translating from Chinese to English, participants were provided with 313 Chinese words. However, due to the semantic similarity between “情感”^1^ and “感情,” which were both commonly translated as “emotion,” only 312 English words were included in the English-Chinese translation direction. This process yielded a total of 625 translation equivalent word pairs. Adhering to the criterion established in previous studies (Tokowicz and Kroll, 2007; Basnight-Brown and Altarriba, 2016), word pairs with only one translation equivalent in each direction were classified as OTPs, while word pairs with more than one translation equivalent in at least one direction were deemed to be MTOTPs.

Next, the control variables, including word concreteness, word frequency, dominant part of speech, word length, orthographic neighborhood density and cognate status, were matched and controlled across translation equivalent word pairs. English word concreteness was determined in compliance with the database from Brysbaert et al. (2014), while the assessment of Chinese words’ concreteness was in line with Wang et al. (2021), entailing 25 graduate students to evaluate with a 5-point rating scale (1 for “abstract,” 5 for “concrete”). With references to the SUBTLEX-US and SUBTLEX-CH (Brysbaert et al., 2012; Cai and Brysbaert, 2010), we obtained the English and Chinese word frequencies and identified noun as the dominant part of speech for selected Chinese and English words. The English Lexicon Project developed by Balota et al. (2007) and a Chinese lexical database assembled by Sun et al. (2018) were drawn on, respectively, to acquire the letter and stroke count for these English and Chinese words. English words’ orthographic neighborhood density was calculated utilizing the Levenshtein distance, sourced from the English Lexicon Project developed by Yarkoni et al. (2008). The *Modern Chinese Dictionary (7th Edition)* (Dictionary Editorial Office of Linguistics Institute of Chinese Academy of Social Sciences, 2016) was also referred to select non-cognate words for controlling the potential influence of cognate status on the results.

In all, 240 translation equivalent word pairs were chosen for the experiment, with 60 OTPs and 60 MTOTPs in each direction. A one-way analysis of variance (ANOVA) was performed in turn on the above properties of experimental materials in four conditions constructed by crossing the two factors of the number of translations (OTP, MTOTP) as well as translation direction (L1–L2, L2–L1). The results demonstrated a significant main effect of the number of translations, *F*(3,236) = 229.04, *p* < 0.001, $\eta_{p}^{2}$ =0.74. Further analysis revealed significant differences in the number of translations between OTPs and MTOTPs in both translation directions [all *p*s = 0.001, *Cohen’s d*(s) ≥ 3.25]. However, no significant differences were found between OTPs or between MTOTPs in two different translation directions. To be precise, for the OTP group, the number of translations for all words was 1 in both translation directions, showing no difference (*Cohen’s d* = 0). The number of translations for L1–L2 and L2–L1 directions in the MTOTP group was calculated as 2.32 and 2.22, respectively (*p* = 0.46, *Cohen’s d =* 0.27). Moreover, there were no statistical differences in the concreteness, frequency, and length of words in both languages separately, nor in the orthographic neighborhood density of English words, across all conditions [all *p*s ≥ 0.09, $\eta_{p}^{2}$ (s) ≤ 0.03].

Guided by the research aim of observing and comparing the masked translation priming effect, we additionally selected 120 English words and 120 Chinese words, which were without semantic association with the target words, as control primes. English control primes were matched in terms of the concreteness, frequency, number of letters, and orthographic neighborhood density with translation primes in OTPs and MTOTPs [all *p*s ≥ 0.29, *Cohen’s d*(s) ≤ 0.14]. Similarly, the concreteness, frequency, and stroke count of Chinese control primes were matched to those of translation primes within both OTP and MTOTP conditions [all *p*s ≥ 0.13, *Cohen’s d*(s) ≤ 0.20].

To achieve equilibrium between the responses of “Yes” and “No” in our lexical decision task, 120 English primes paired with Chinese nonwords and 120 Chinese primes coupled with English nonwords were chosen to serve as fillers. The Chinese nonwords were all meaningless words which were made up of two characters, and matched with those real Chinese targets in the stroke count (*p* = 0.94, *Cohen’s d =* 0.01). Generated and obtained from the ARC Nonword Database (Rastle et al., 2002), the English nonwords in this study were also matched with the real English targets in the letter count (*p* = 0.96, *Cohen’s d =* 0.01).

Finally, four presentation lists, two for each direction, were composed to counterbalance the experimental materials based on the equivalence and relatedness between prime words and target words. Thus, each target word occurred only once per list, either with the translation prime condition (e.g., “情感”–“emotion” “style”–“风格”) or the unrelated prime condition (e.g., “明天”–“emotion” “effort”–“风格”).

### 2.3 Procedure

In our study, the Gorilla Experiment Builder (Anwyl-Irvine et al., 2020) was utilized to perform the experiment. We primarily recruited Chinese learners from English-speaking countries like the United Kingdom, the United States, and Canada, as their native language background is more representative. However, the pool of native English-speaking Chinese learners is quite limited, and among those from typical English-speaking countries, this number is even smaller, with most being beginners. To address this, we followed the approach of Soo and Monahan (2023) and primarily recruited participants from universities in China, as well as through Prolific,^2^ a professional platform for recruiting participants. Ultimately, a total of 23 participants were recruited via the former method, of which 16 participants from Beijing completed the experiment one-on-one under the supervision of researchers. Although G*Power measurement indicates that 16 participants are sufficient to achieve a statistical power of 0.80, we opted to increase the number of participants further to enhance statistical power. Some participants, located in cities such as Harbin and Xi’an, or those who returned to their home countries after the final exams, conducted the experiments remotely under the supervision of the researchers through screen sharing via Tencent Meeting, following approaches similar to those adopted in the studies by Wang Y. et al. (2023) and Hu and Zhao (2023). Based on recommendations from the Prolific team and insights from de Bruin et al. (2023), we first performed a pre-screening when recruiting participants through Prolific to ensure that all participants were proficient Chinese learners whose native language was English. Out of 178,310 available participants on Prolific, fewer than 25 met the eligibility criteria. After inviting them, only one took part in the formal experiment.

Some researchers have advocated that high-quality data can be obtained from online masked priming experiments in which participants are recruited through Prolific (Angele et al., 2023; Scrimshire et al., 2023). In addition, a growing body of studies has successfully utilized the Gorilla Experiment Builder to collect data in an unmonitored state (Soo and Monahan, 2023; Gosselin and Sabourin, 2023; de Bruin and Xu, 2023), establishing the viability and acceptance of this approach in the research community. Nevertheless, we took measures to ensure the data collected in the unmonitored condition was of high quality, specifically by implementing an attention check task based on the approach described by Chaouch-Orozco et al. (2024). Participants were instructed to press the “B” key within 2 s at the commencement of the task instructions, with checks conducted approximately every 15 trials. This participant in the unmonitored condition achieved 100% accuracy on the attention checks and completed the experimental task with a high accuracy rate of 95.42%, indicating that the participant approached the task with diligence. Additionally, with reference to Fricke (2022), we restricted the type of devices participants could use to take part in the experiment. Participants were required to use a desktop or laptop computer, rather than phones or tablets, to maximize the likelihood that they completed the experiment seated and in the least intrusive environment.

**Figure 1:**
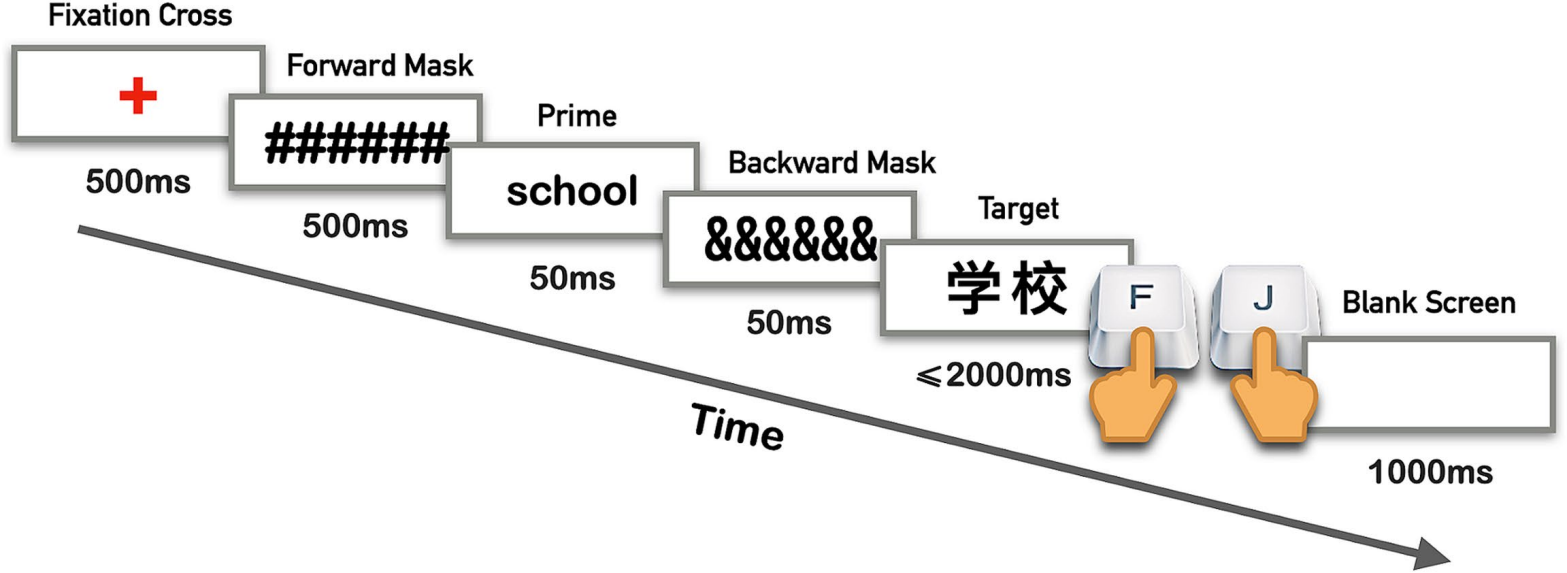
The presentation sequence and timing of items in the experiment.

Participants needed to complete a lexical decision task, attempting to make accurate and prompt decisions about whether the target words were real words or nonwords. Responses were indicated by pressing down either the “J” or “F” key, with key assignments for real words counterbalanced among participants. The experimental procedure was adapted from previous studies such as Chen et al. (2014) and McPhedran and Lupker (2021). During the onset of each experimental trial, the fixation cross “+” appeared for 500 ms, after which a row of hash marks was displayed for another 500 ms as a forward mask. Subsequently, a prime word emerged for 50 ms and then followed by a row of ampersands, serving as a backward mask, which also lasted 50 ms. Next, a target word was presented to participants until they responded, with a maximum duration of 2000 ms. A blank screen would appear over 1,000 ms after each trial, signaling the interval between trials (as illustrated in Figure 1). Prior to the formal experiment, participants engaged in at least 34 practice trials, consisting of 17 English-Chinese and 17 Chinese-English word pairs, to ensure they were familiar with the procedure.

Following Schoonbaert et al. (2009), Wang (2013), Ferré et al. (2017), Lee et al. (2018), and Li et al. (2024), each prime word is exhibited for 50 ms. As far as the studies of the bilingual masked priming effect are concerned, an SOA of 100 ms is considered appropriate (Altarriba and Basnight-Brown, 2007). However, a 100 ms duration solely dedicated to prime presentation could lead to conscious processing (Jiang, 2023). To address this, we followed Schoonbaert et al. (2009) and Ferré et al. (2017), attaching a backward mask lasting 50 ms after each prime presentation. Furthermore, we chose different symbols as the forward and backward masks in this study. This combination of manipulations helps to prevent the prime word’s visibility and to enhance the time allotted for its processing (Xia and Andrews, 2015).

## 3 Results

All participants achieved an accuracy rate above 83%, with none falling below 70% (Chen et al., 2022); therefore, the final analysis included data from all 24 participants. With regard to data analysis, after excluding the data stemming from practice trials and nonword items, 4.27% of the responses generated by 24 participants as incorrect responses were further discarded. Outliers in RTs that deviated from the condition mean by ±3 *SDs* were also removed, accounting for 1.63% of the total responses. RTs were log-transformed as done by de Bruin et al. (2023) to normalize the distribution. Analyses of RTs and accuracy rates were conducted in R (version 4.4.1) utilizing (generalized) linear mixed-effects models (Baayen et al., 2008) with lmer and glmer functions of the lme4 package (Bates et al., 2015). Following previous studies, including those by McPhedran and Lupker (2021), Lim and Christianson (2023), and Li et al. (2024), the fixed-effects factors in our model comprised the number of translations, translation direction, prime type, and cloze test scores (L2 Chinese proficiency), and subjects, primes, and targets were included as random effects. We centered and standardized the cloze test scores as a continuous variable and checked the collinearity among the variables included in the model, as all variance inflation factor (VIF) values were below 3.14, which is well below the threshold of 5 (Craney and Surles, 2002; Momenian et al., 2021). We referred to Wu (2019) and Zhou and Jiang (2023) in removing variables through a backward stepwise selection procedure, and to compare models using the anova() function. The model most compatible with our data was selected based on the Akaike information criterion (AIC). Building upon Hao et al. (2022, 2024) and Cui et al. (2024), we analyzed the main and interaction effects of the variables with the Anova() function, and the contrast() function was used to carry out the *post hoc* analyses. The descriptive statistics for mean RTs and accuracy rates across a variety of experimental conditions are depicted in Table 2.

**Table 2:** Mean RTs (ms) and accuracy rates (%, in parentheses) under all experimental conditions.

Number of translations	Translation direction	Prime type	
		Translation	Unrelated
OTPs	L1–L2	773.57 (95.14)	887.61 (91.67)
	L2–L1	558.98 (99.31)	571.57 (99.17)
MTOTPs	L1–L2	824.39 (94.31)	907.45 (89.03)
	L2–L1	577.63 (98.75)	578.48 (98.47)

### 3.1 The influence of translation direction on masked translation priming effect

In the RT analysis, the interaction involving translation direction and prime type was exhibited to be significant, *χ^2^*(1) = 105.69, *p* < 0.001, implying that the priming magnitude was significantly larger in the L1–L2 direction (*M* = 98.47 ms) compared to the reverse direction (*M* = 6.70 ms). Further analysis showed significantly faster RTs for targets preceded by translation primes than those preceded by unrelated primes in the L1–L2 direction, *β* = 0.12, *SE* = 0.01, *z* = 15.50, *p* < 0.001, while no significant difference was observed between the translation and unrelated prime conditions in the L2–L1 direction, *β* = 0.01, *SE* = 0.01, *z* = 1.45, *p* = 0.148.

In the accuracy analysis, the interaction involving translation direction and prime type was not significant, *χ^2^*(1) = 2.21, *p* = 0.137.

### 3.2 The influence of number of translations on masked translation priming effect

A significant interaction between the number of translations and prime type was also evident in the results for the RTs, *χ^2^*(1) = 4.87, *p* = 0.027, suggesting that the amount of priming in the OTP condition (*M* = 59.47 ms) was significantly larger than that in the MTOTP condition (*M* = 37.21 ms). Further analysis indicated that target words preceded by their translation primes elicited significantly faster RTs compared to those preceded by unrelated primes when presenting either OTPs (*β* = 0.07, *SE* = 0.01, *z* = 10.26, *p* < 0.001) or MTOTPs (*β* = 0.05, *SE* = 0.01, *z* = 7.11, *p* < 0.001).

No significant interaction was observed between the number of translations and prime type for the accuracy data, *χ^2^*(1) = 0.40, *p* = 0.529.

### 3.3 The impacts of translation direction on the priming effect differ under OTP and MTOTP

**Figure 2:**
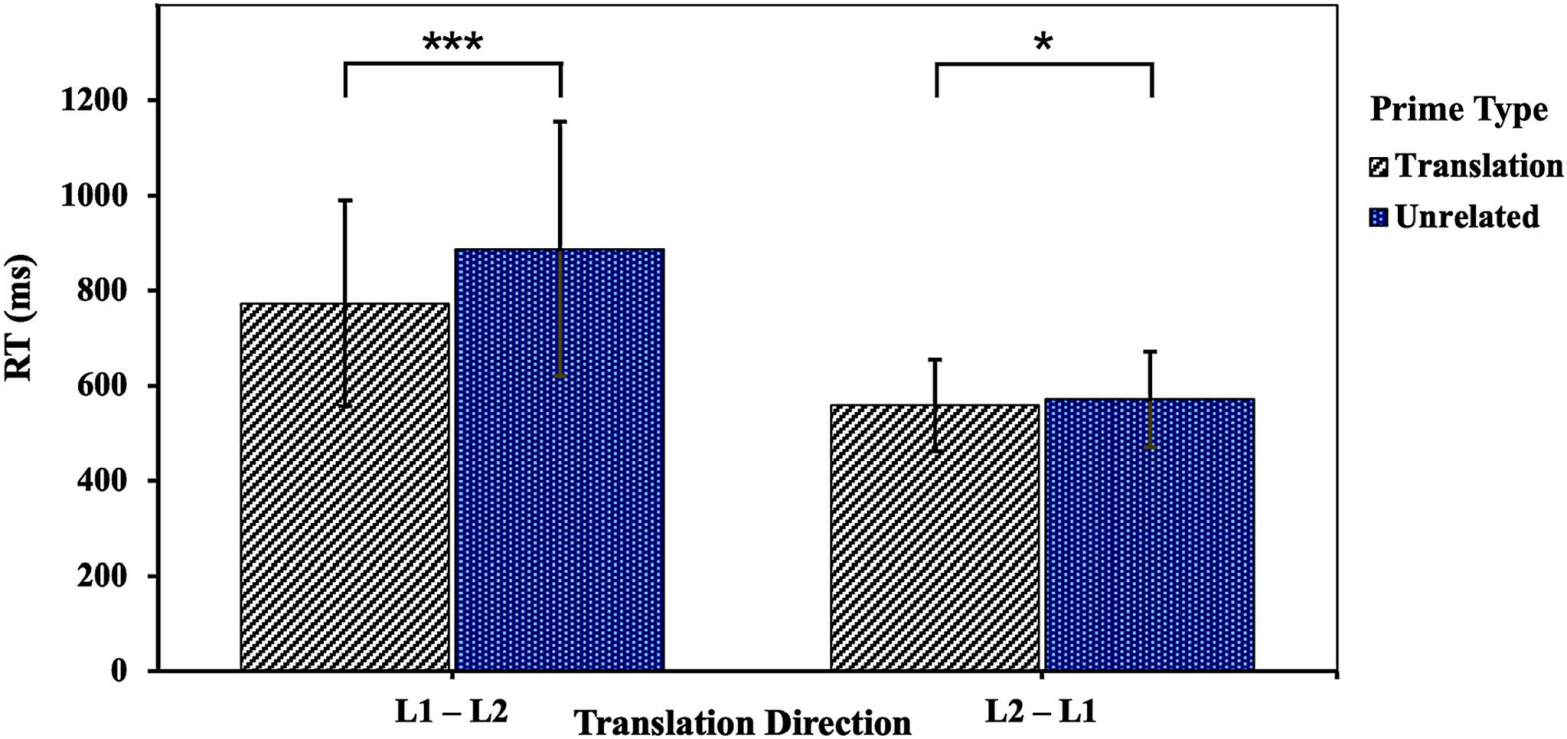
The influence of translation direction on the priming effect under OTP condition. ***p < 0.001; *p < 0.05.

**Figure 3:**
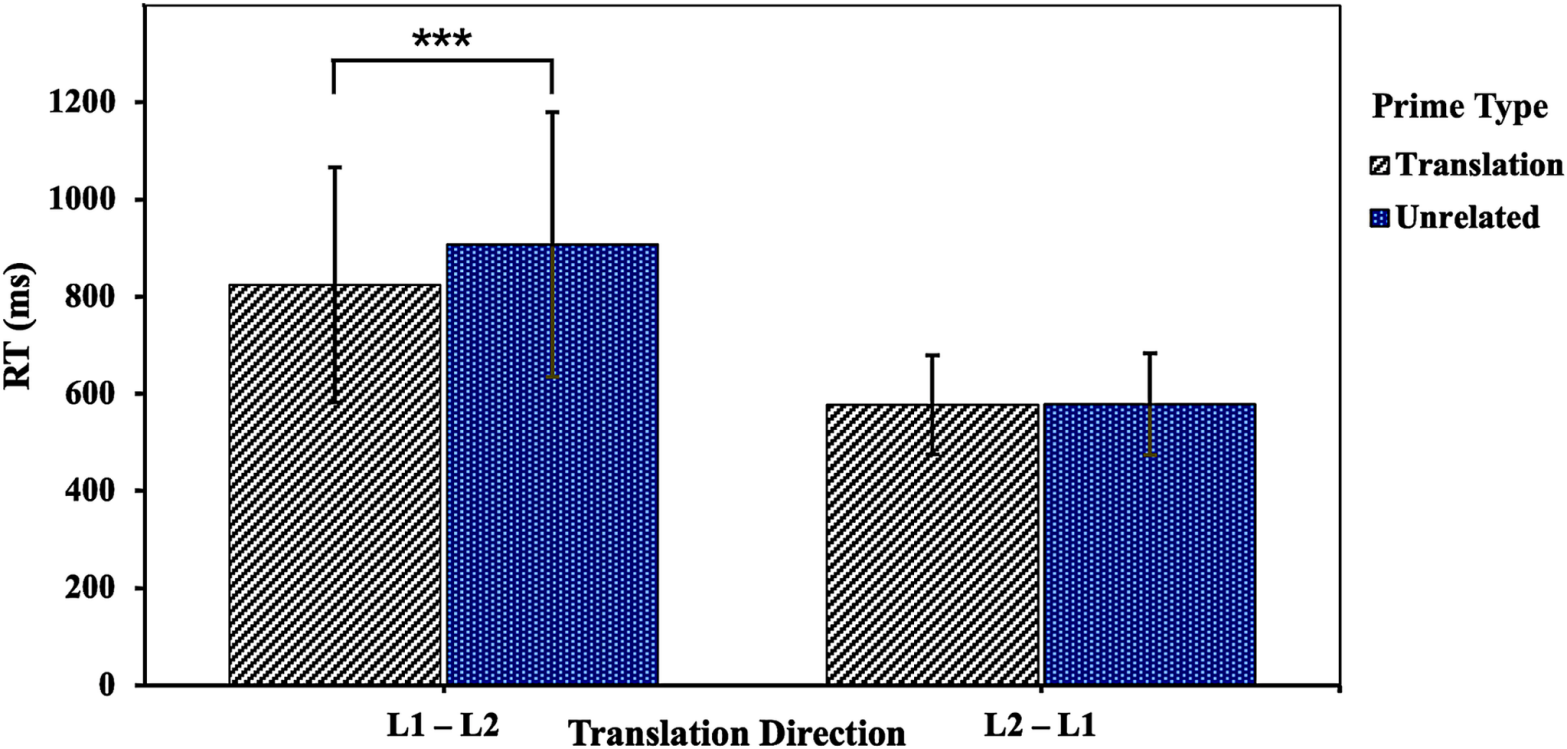
The influence of translation direction on the priming effect under MTOTP condition. ***p < 0.001.

No significant three-way interaction among the number of translations, translation direction and prime type was found in RT and accuracy analyses (both *ps* > 0.05). To clarify whether the influence of translation direction on the priming effect differs for OTPs and MTOTPs, we referred to the data analysis methods of Ferré et al. (2017) and Hu and Zhao (2023) in analyzing the experimental data for each condition separately. The RT analysis revealed that, for OTPs, the interaction between translation direction and prime type was significant, *χ^2^*(1) = 60.62, *p* < 0.001, indicating that the priming effect was much greater in the L1–L2 direction (*M* = 114.04 ms) than in the reverse direction (*M* = 12.59 ms). Further analysis showed that RTs were shorter when targets were preceded by translation primes than when they were preceded by unrelated primes either in L1–L2 (*β* = 0.13, *SE* = 0.01, *t* = 12.58, *p* < 0.001) or L2–L1 direction (*β* = 0.02, *SE* = 0.01, *t* = 1.98, *p* = 0.048) (as visualized in Figure 2). A significant interaction for MTOTPs between translation direction and prime type occurred as well, *χ^2^*(1) = 45.00, *p* < 0.001, suggesting that the amount of priming in the L1–L2 direction (*M* = 83.06 ms) was greater than in the L2–L1 direction (*M* = 0.86 ms). However, targets preceded by translation primes elicited significantly faster RTs than those preceded by unrelated primes only in the L1–L2 direction (*β* = 0.10, *SE* = 0.01, *t* = 9.32, *p* < 0.001), while no significant difference was observed in the reverse direction (*β* = 0.00, *SE* = 0.01, *t* = 0.12, *p* = 0.905) (see Figure 3).

However, the accuracy data analysis demonstrated no significant interaction between translation direction and prime type neither for OTPs, *χ^2^*(1) = 0.52, *p* = 0.473, nor for MTOTPs, *χ^2^*(1) = 2.00, *p* = 0.157.

In the context of translation priming effect research, researchers tend to place emphasis on RTs (Gao and Liu, 2016; Chaouch-Orozco et al., 2024), as accuracy rates are often regarded as insufficiently sensitive to experimental manipulations. This is further supported by meta-analyses and literature reviews in the field, which have solely concentrated on RTs (Wen and van Heuven, 2017b; Davis and Kim, 2021; Jiang, 2023). Aligned with Chaouch-Orozco et al. (2024), the accuracy data analysis in this study revealed only main effects of prime type, *χ^2^*(1) = 21.64, *p* < 0.001, and translation direction, *χ^2^*(1) = 53.45, *p* < 0.001, with the accuracy rates being significantly higher for the targets preceded by translation primes (2.29% difference) and in the L2–L1 direction (6.39% difference), as well as a marginally significant main effect of the number of translations, *χ^2^*(1) = 3.22, *p* = 0.073, with a higher accuracy rate for OTPs (1.18% difference).

It was also shown that the effects of translation direction and the number of translations on prime type were not modulated by the cloze test scores (all *p*s > 0.05). Therefore, cloze test scores did not influence the main findings pertaining to our research questions. Within both the RT and accuracy analyses, the interaction effects involving cloze test scores were observed only between it and translation direction (both *ps* < 0.001). To explore whether the processing of participants with relatively high and low proficiency both exhibits the effect of translation direction. Meanwhile, drawing on the methods of Lim and Christianson (2023), we first categorized participants into two groups based on their cloze test scores (*Mdn* = 23): those scoring above 23 were classified as relatively high proficiency, and those scoring 23 or below as relatively low proficiency. This classification ensured a significant difference in the two groups’ scores (*p* < 0.001). Ultimately, it was found that the differences in RTs and accuracy rates in both directions were greater for the relatively low proficiency group. Further analyses revealed that both relatively high and low proficiency groups processed faster and more accurately in the L2–L1 direction.

All in all, the experimental results revealed a significant masked translation priming effect. Notably, this effect was affected by translation direction and the number of translations. Regarding translation direction, the priming magnitude was significantly greater in the L1–L2 direction, with the significant priming effect observed only in this direction. As for the number of translations, participants responded significantly faster to the targets preceded by their translations than to those preceded by unrelated primes whether with OTPs or MTOTPs; however, the priming effect was greater in the OTP condition. Moreover, the influence of translation direction on the priming effect was also shown to be modulated by the number of translations. To elaborate, the priming effect was significantly larger in the L1–L2 direction than in the L2–L1 direction regardless of OTPs or MTOTPs. But when participants responded to OTPs, significant priming effects were seen in both translation directions, while for MTOTPs, significant priming effect was found merely in the direction of L1–L2, with no effect detected in the reverse direction.

## 4 Discussion

Based on the above results and using relevant models of the bilingual mental lexicon, we first shed light on Question (2) independently, and then proceed with a focused discussion combining Question (1) and Question (3). This explication sequence is driven by the fact that investigating the influence of the number of translations in Question (2) lays the foundation for the subsequent inquiry in Question (3). Also, both Question (1) and Question (3) involve examining how translation direction impacts the masked translation priming effect.

### 4.1 The influence of number of translations on masked translation priming effect

This study reveals that the priming effect for OTPs is significantly more pronounced than that for MTOTPs, indicating differences in semantic representation between these two types of translation pairs in the brains of bilingual individuals. Differing from most existing studies (Schoonbaert et al., 2009; Wang and Zhang, 2013; Ferré et al., 2017) on masked translation priming effects that treated the number of translations as a control variable, our experimental materials encompassed both OTPs and MTOTPs, allowing us to probe how the number of translations influences the masked translation priming effect in a direct way. Additionally, based on the findings of prior studies which have found a processing advantage for OTPs (Laxén and Lavaur, 2010; Wang and Wang, 2019; Basnight-Brown et al., 2020), we further combined the number of translations with the masked translation priming effect to inquire into the detailed and intricate interaction between the two variables, and identified a priming advantage for the OTPs.

The DCFM provides a plausible explanation for the results of this study. The magnitude of cross-language priming is closely associated with the amount of conceptual overlap between the words in L1 and L2, which could be embodied as the extent to which semantic features are shared between primes and targets (Schoonbaert et al., 2009). As the connection strength grows with a greater degree of shared semantic features between words, the activation intensity becomes stronger, leading to a more pronounced priming effect. Concerning the number of translations, there is only one translation for a word in an OTP. This translation shares nearly all the semantic features with the given word. By contrast, for a word in MTOTPs that possesses multiple translations, each translation shares only partial semantic features with the word (Laxén and Lavaur, 2010). Compared to MTOTPs, OTPs display a higher proportion of shared semantic features, indicating a stronger priming effect.

### 4.2 The influence of translation direction on masked translation priming and its differences between OTP and MTOTP conditions

Consistent with Wang’s (2013) findings on unbalanced English-Chinese bilinguals, this study identified a significant effect of translation direction on masked translation priming. Generally, a significant priming effect was observed only in the L1–L2 direction but was absent in the reverse direction. Although the bilinguals invited to go through our experiment have undergone systematic Chinese language learning in China and reached an intermediate-advanced level of proficiency in Chinese as an L2, they are still unbalanced bilinguals. On the basis of the RHM, for unbalanced bilinguals, the conceptual representation should be connected more robustly with their L1 words than L2 words. When L1 primes are presented, L1 lexical representations can pre-activate the shared conceptual representation with L2 words, which in turn facilitates access to the L2 lexical representations, aiding in L2 target word recognition and thereby generating the priming effect in the direction from L1 to L2. Conversely, the connections between L2 words and the conceptual system are weaker, rendering L2 primes less likely to pre-activate the shared conceptual representation with L1 words. As a consequence, the processing of L2 primes does not effectively assist in the priming for L1 targets.

Notwithstanding that we set the SOA in this study to 100 ms, which varied from Wang (2013), where the SOA was 50 ms, it is still difficult to observe a significant L2–L1 priming effect. With an SOA of 100 ms, Schoonbaert et al. (2009) came to a different finding than we did, obtaining a priming effect in the L2–L1 direction in the case of unbalanced Dutch-English bilinguals. Also, Lee et al. (2018) found priming in the same direction for unbalanced Korean-English bilinguals with an SOA of 150 ms. Considering the differences in writing systems, we postulate that different-script language pairs necessitate a longer SOA to detect a priming effect in the L2–L1 direction for unbalanced bilinguals. Future studies could consider using a longer SOA.

Even if the participants reached the intermediate-advanced level of proficiency in L2 Chinese and the SOA was set at 100 ms, no priming effect was observed in the L2–L1 direction in this study. However, it is worth mentioning that the impact of translation direction on masked translation priming diverged between OTPs and MTOTPs. For OTPs, both directions exhibited the priming effects, whereas for MTOTPs, the priming effect existed only in the L1–L2 direction.

In light of the DCFM and Sense model, L1 words are expected to contain relatively more extensive semantic information than those in L2 for unbalanced bilinguals, indicating the discrepancy in the extent to which shared semantic features are activated by words in two languages (Finkbeiner et al., 2004; Schoonbaert et al., 2009). When an L1 word primes its translation equivalent in L2, the prime word, with its richer semantic representation, may be able to cover most or all of the target word’s semantic information, engendering greater semantic overlap between the L1 prime and the L2 target. This allows the L1 word to activate more shared semantic features than the L2 word, resulting in a stronger priming effect. For MTOTPs, the correspondence between a word in the source language and its translations in the target language is characterized by a one-to-many pattern (Schwieter and Prior, 2020). The difficulty in processing word pairs escalates with the increasing number of translations, which highlights the advantage of a richer semantic representation in L1 compared to L2. This means that primes in L1 are supposed to activate more semantic features, thereby generating a significant L1–L2 priming effect. Our findings align with those of Ferré et al. (2017), who also found a priming advantage for the MTOTPs in the direction of L1–L2. On the other hand, OTPs have a one-to-one mapping (Lee et al., 2023). Given the high degree of semantic overlap, it is conceivable that L2 words might activate L1 translations as effectively as L1 words activate L2 translations, thus producing a significant L2–L1 priming effect. It is in agreement with the findings of Schoonbaert et al. (2009).

Using MTOTPs as experimental materials, Wang and Zhang (2013) detected a notable priming effect in the direction of L2–L1, which is at odds with the findings of our study. As argued by the RHM mentioned earlier, this discrepancy may be ascribed to the higher L2 proficiency of participants in their study, who were highly proficient Chinese-English bilinguals having achieved passing scores on the Test for English Majors Band 8 (TEM-8), the highest-level test to measure the English proficiency of Chinese students majoring in English.

The study reveals a significant masked translation priming effect, indicating a conceptual system shared by both languages of unbalanced English-Chinese bilinguals (Ferré et al., 2017; Bu and Lu, 2021; Wang X. et al., 2023). The notable priming effect observed solely in the L1–L2 direction implies an incomplete sharing of conceptual representation across an unbalanced bilingual’s two languages (Chen et al., 2017). Moreover, the priming effect for OTPs is significantly greater than that for MTOTPs, suggesting differences in semantic representation between these two categories of translation pairs for bilinguals—words in OTPs exhibit a more complete shared conceptual representation compared to those in MTOTPs. Furthermore, the priming effect for OTPs is evident in both translation directions, which implies that the priming asymmetry is reflected in the priming size; the priming effect for MTOTPs only occurs in the L1–L2 direction, indicating that the asymmetry manifests with respect to translation direction. Thus, there are certain differences in lexical representation and organization patterns between OTPs and MTOTPs for unbalanced English-Chinese bilinguals. For an OTP, the larger semantic overlap may help to establish conceptual connections in the L2–L1 direction, albeit weaker compared to the L1–L2 direction; while, for an MTOTP, connections in the L2–L1 direction are typically achieved more on the lexical rather than the conceptual way.

This research has certain limitations. First, although it examines the effects of translation direction and the number of translations on the masked translation priming effect, a more in-depth and comprehensive investigation is recommended by including participants with a broader range of L2 proficiency levels and integrating additional experimental factors, such as SOA, with the variables examined in this study. Second, three participants in this study reported having another native language in addition to English. Albeit they had attained intermediate-advanced proficiency in Chinese, attended at least 4 h of Chinese classes daily on weekdays, and primarily used both English and Chinese for communication with peers and instructors, we did not systematically investigate or record the amount of time they spent using each language daily. In future studies, apart from participants’ language proficiency, it is also important to clarify the differences in the duration of their usage of various languages as well as to ensure more strictly the exclusivity of participants’ native language backgrounds. Third, due to the relatively limited number of Chinese learners with English as their native language and the intermediate-advanced level language proficiency requirements in this study, the data collection process involved both one-on-one monitoring of participants completing the experiment and online recruitment via the experimental platform. Although we took various measures to ensure that participants approached the experiment with due diligence, it may be preferable to adopt only one way of participation to minimize the potential impact caused by the participation mode.

## 5 Conclusion

In summary, the masked translation priming effect was found to be significantly influenced by translation direction and the number of translations. Furthermore, the influence of translation direction on the priming effect differs between the conditions of OTPs and MTOTPs. In essence, acquiring an L2 refers to a process that the established L1 system interacts with a new language representation system (Kim et al., 2016); on that account, another potentially fruitful avenue for future research is to integrate computational models, like the Developmental Lexicon II model proposed by Zhao and Li (2010, 2013, 2022), to observe the development process of L2 vocabulary learning and the dynamic interaction taking place between the lexicons in two languages. Even though there are still several unanswered questions, the current study offers contributions to the field related to the bilingual lexical representation and organization as a pioneering systematic exploration of how the number of translations influences the masked translation priming effect and modulates priming asymmetry among unbalanced English–Chinese bilinguals. Moreover, this study also focuses on different writing systems, providing new empirical evidence from unbalanced English–Chinese bilinguals for going into the cross-language priming effect.

## Data Availability

The datasets presented in this study can be found in online repositories. The names of the repository/repositories and accession number(s) can be found at: DOI 10.17605/OSF.IO/SM2RH.

## Footnotes

^1^We used “情感” rather than “感情” in our experimental materials. This choice was based on participants’ responses. Specifically, in the Chinese-English translation direction, a greater proportion of participants produced the English word “emotion” when “情感” as opposed to “感情” was presented; and in English-Chinese translation direction, a higher proportion of participants responded with “情感” compared to those who wrote “感情” when presented with “emotion.”

^2^ https://prolific.co/
